# Characteristics and productivity of the chiropractic workforce of the Veterans Health Administration

**DOI:** 10.1186/s12998-022-00429-1

**Published:** 2022-04-11

**Authors:** Kelsey L. Corcoran, Douglas R. Peterson, Xiwen Zhao, Eileen A. Moran, Anthony J. Lisi

**Affiliations:** 1 Pain Research, Informatics, Multimorbidity and Education (PRIME) Center, VA Connecticut Healthcare System, 950 Campbell Avenue, West Haven, CT USA; 2 Yale Center for Medical Informatics, Yale School of Medicine, 300 George Street, New Haven, CT USA; 3 Office of Productivity, Efficiency and Staffing, VA Central Office, 810 Vermont Ave NW, Washington, DC USA; 4 Yale Center for Analytical Sciences, Yale School of Public Health, 300 George Street, New Haven, CT USA

**Keywords:** Chiropractic, Workload, Integrative medicine

## Abstract

**Background:**

Increasingly, integrated healthcare systems such as the United States Veterans Health Administration (VHA) are employing chiropractors. However, little is known about chiropractor employee clinical productivity which may be important for resource planning and monitoring care delivery. With its history of delivering chiropractic care and its enterprise-level assessment metrics, the VHA is an ideal setting to study a chiropractic workforce. We aim to assess characteristics of chiropractors employed by the VHA and explore associations between these characteristics and clinical productivity.

**Methods:**

This was a cross-sectional and serial analyses of VHA administrative data. Characteristics of the chiropractor workforce were evaluated from fiscal year (FY) 2016 to FY2019. Productivity was calculated using the VHA productivity measure, the quotient of an individual’s total work relative value units (wRVUs) per FY divided by the direct clinical full-time equivalent (FTE) worked. A multivariable regression model was used to analyze the association between productivity and characteristics of the chiropractor and VHA facility.

**Results:**

From FY2016 to FY2019, the number of chiropractor employees increased from 102 to 167. In FY2019, the typical chiropractor employee was male, white, and 45.9 years old with 5.2 years of VHA experience. In FY2019, the VHA chiropractor workforce was 25.1% female, 79% white, and 20.4% Veteran. The productivity measure of a chiropractor was 3040 in FY2019. A higher facility complexity measure, presence of 3 chiropractor employees at a facility, and older age of the providers were the only characteristics studied that had a significant impact on productivity after adjusting for other covariates.

**Conclusion:**

Provider characteristics and productivity metrics of the VHA chiropractor employee workforce are presented. The productivity measure provides an initial benchmarking that may be relevant to future modeling of chiropractor personnel in VHA and other healthcare systems.

## Background

The Veterans Health Administration (VHA) is the largest integrated healthcare system in the US and has offered chiropractic care on-site at an increasing number of VHA facilities since the service was first introduced in 2004 [1–4]. Through ongoing program development, and in response to the Consolidated Appropriations Act, 2018, VHA is expanding the number of chiropractic clinics at its medical centers across the country and including chiropractic care as a preventive healthcare benefit for Veterans [5]. It is likely that part of what is driving the trend of increased integration of chiropractic care into larger healthcare systems like VHA is an increase in the need for nonpharmacological pain management modalities in light of the devastation of the opioid crisis and prior opioid prescribing patterns in the US [6–10]. Therapies that chiropractors provide are recommended as frontline treatments by several clinical practice guidelines for musculoskeletal pain, including the American College of Physicians’ low back pain guidelines [11–14]. This has contributed to the recommendation to incorporate chiropractic care as a component of musculoskeletal pain management and the stepped care model of pain management in VHA [15, 16]. There is also a growing body of evidence demonstrating an association between chiropractic services use and decreased prescription opioid receipt [17–25].

According to the National Board of Chiropractic Examiners’ 2020 Practice Analysis, only 5.4% of chiropractors report having hospital privileges [26]. While chiropractic services are growing within larger healthcare systems including VHA, chiropractors are still a relatively new provider type at most hospitals and medical centers where they are employed [1, 27]. There is currently no published literature about the magnitude of clinical productivity of chiropractor employees in integrated healthcare systems. However, knowledge of the productivity of clinical services can be important for resource planning and monitoring those services [28].

The VHA is an ideal setting to examine chiropractor productivity in the US since it has been an established service in this hospital system for over 15 years. The objective of this paper is to report on the characteristics and productivity of the VHA chiropractic workforce, and explore associations between these factors.

## Methods

### Study design/population

Cross-sectional and serial analyses of VHA administrative data were conducted to evaluate VHA chiropractor employee characteristics and productivity. The study population included all chiropractor employees of the VHA from October 1, 2015 to September 30, 2019. This study was reviewed and approved by the Institutional Review Board of the Veterans Affairs Connecticut Healthcare System.

### Data sources and variables

Data were obtained from multiple sources that are accessible from the United States Department of Veterans Affairs’ VHA Service Support Center. Data were reported based on fiscal year (FY) of the US government, which runs from October 1 of the prior year to September 30 of the year indicated.

The productivity measure is the primary outcome of the productivity analysis and a product of the VHA Office of Productivity, Efficiency, and Staffing (OPES). The productivity measure is calculated by dividing the annual work relative value units (wRVUs) of an individual chiropractor by their direct clinical full-time equivalent (FTE) worked. The annual wRVUs of a clinician is derived from procedures performed and coded for using Current Procedural Terminology® (CPT®) codes. Each procedure coded using a CPT® code has an assigned wRVU from the Medicare Physician Fee Schedule [28, 29]. WRVUs are set for each service based on an agreed upon measure of the technical skill, time, and cognitive effort required by the physician for each service [29]. The FTE of an employee represents the work assignment and is a number of 1 or less with a 1 representing a full-time employee. Direct clinical FTE worked is the total FTE of a chiropractor labor mapped to clinical duties and adjusted for time off.

Age, sex, race/ethnicity, Veteran status, years of VHA service, and salary information were obtained from the Human Resources cube for all employees classified as a chiropractor. Sex from FY2016 to FY2019 is recorded as male or female. Race/ethnicity was reported from the race/ethnicity categories of white, Black, Asian, Hispanic, or other.

Variables collected about the chiropractic practice of each chiropractor included unique patients, total visits and mean wRVUs per visit. Unique patients were counted based on Social Security number to avoid duplication.

Facility characteristics included medical complexity grouping (MCG) and staffing levels. The MCG of each VHA facility is based on a VHA Facility Complexity Model and a product of OPES [30]. This MCG index ranks facilities as 1a, 1b, 1c, 2 or 3, with 1a representing the most complex facilities and 3 representing the least complex facilities using this rating system [30]. The MCG is modelled based on measures of the patient population, clinical services complexity, education, and research [30]. Staffing levels including FTE employee values for chiropractors, administrative support staff, and clinical support staff are from the Specialty Provider Productivity Cube, a database maintained by OPES. Administrative support staff (i.e. medical support assistants) and clinical support staff (i.e. nurses) were considered in the Administrative Support Staff FTE per Chiropractor FTE and Clinical Support Staff FTE per Chiropractor FTE calculations, respectively, based on the amount of their effort labor mapped to supporting the chiropractic clinic and divided by the total chiropractor clinical FTE at their facility.

### Analysis

The characteristics of the chiropractic workforce were investigated using serial analysis from FY2016-FY2019.

To analyze clinical productivity of the workforce, only those individuals with complete data for FY2019 were included. Descriptive statistics were used to summarize subject characteristics, and included mean, standard deviation, median, and range if continuous, or count and percentage if categorical. The association between each characteristic and productivity measure are analyzed using univariable linear regression models. Variables that were significantly associated with productivity, as well as characteristics studied in previous literature about clinical productivity, were entered into a multivariable regression model. Statistical significance was set a priori at α = 0.05. All analyses were conducted in R (R Core Team 2020).

## Results

By FY2019, there were 167 chiropractor employees from 81 individual VHA facilities. The characteristics of the VHA chiropractor employee workforce from FY2016 to FY2019 are presented in Table 1.

**Table 1:** VHA chiropractor employee workforce trends

FY	2016	2017	2018	2019
Total DCs	102	107	131	167
FTE	92.2	97.4	122.5	159.2
Female DCs	20 (19.6%)	21 (19.6%)	25 (19.1%)	42 (25.1%)
White DCs**	90 (88.2%)	94 (87.9%)	114 (87.0%)	132 (79.0%)
Veteran DCs	19 (18.6%)	21 (19.6%)	31 (23.7%)	34 (20.4%)
Mean age, years	46.6	47	46.5	45.9
Mean years in VHA	4.6	5.2	5.4	5.2
Mean annual salary	$99,980	$101,998	$104,739	$104,959

*VHA* Veterans Health Administration, *DC* doctor of chiropractic, *FY* fiscal year, *wRVU* work relative value unit, *MCG* Medical Complexity Grouping, *FTE* full-time equivalent

^**^Based on race/ethnicity categories: white, Asian, Black, Hispanic, or other

The sample used for the FY2019 productivity analysis is presented in Table 2 and included 161 unique individuals with complete data (2 individuals missing Normal Scheduled FTE were excluded). In FY2019, the mean productivity measure for a chiropractor employee was 3040 wRVUs/FTE and the median was 2890 wRVUs/FTE. The multivariable model presented in Table 3 found that a higher facility complexity measure, presence of 3 chiropractor employees at a facility, and older age of the providers were the only characteristics studied that had a statistically significant association with the productivity measure when controlling for other covariates.

**Table 2:** VHA chiropractor sample used in the FY2019 productivity analysis

Variable	Total
N	161
Productivity measure (wRVUs/FTE)	3040 (1220)
**Age, years**	
< 40	53 (32.9%)
40–49	46 (28.6%)
50–59	35 (21.7%)
60+	27 (16.8%)
**Sex**	
Female	36 (22.4%)
Male	125 (77.6%)
Years served in VHA	5.52 (4.98)
Normal scheduled FTE	0.827 (0.293)
Unique patients	433 (244)
Patient visits	1750 (980)
Mean wRVUs per visit	1.24 (0.387)
**Number of DCs**	
1	31 (19.3%)
2	63 (39.1%)
3	28 (17.4%)
3+	39 (24.2%)
Administrative support staff FTE per DC FTE	0.294 (0.340)
Clinical support staff FTE per DC FTE	0.155 (0.246)
**MCG**	
1a-High Complexity	58 (36.0%)
1b-High Complexity	30 (18.6%)
1c-High Complexity	32 (19.9%)
2-Medium Complexity	19 (11.8%)
3-Low Complexity	22 (13.7%)
**Fulltime employee**	
No	49 (30.4%)
Yes	112 (69.6%)

*VHA* Veterans Health Administration, Mean (SD) for continuous variables, *FY* fiscal year, *wRVU *work relative value unit, *DC* doctor of chiropractic, *FTE* full-time equivalent, *MCG* medical complexity grouping

**Table 3:** Regression models of chiropractors’ productivity measures

Variable	Univariable				Mutivariable			
		95% CI				95% CI		
	Coefficient	LCI	UCI	*P*	Coefficient	LCI	UCI	*P*
**Age group (ref: < 40)**				< 0.001				0.1
40–49	262.43	−201.60	726.45	0.27	37.12	−427.65	501.89	0.87
50–59	546.73	45.18	1048.27	0.03	324.49	−199.34	848.32	0.22
60 +	1107.33	562.87	1651.79	< 0.001	657.29	86.09	1228.49	0.02
**Sex**								
Male vs. Female	248.41	−205.94	702.77	0.28	57.75	−383.47	498.98	0.80
**Years served in VHA**								
	52.05	14.64	89.45	0.01	13.85	−26.36	54.06	0.50
**Number of unique patients**								
	1.90	1.17	2.62	< 0.001	0.59	−0.71	1.89	0.37
**Patient visits**								
	0.50	0.32	0.68	< 0.001	0.29	−0.03	0.61	0.07
**Number of DCs per facility (ref: DC = 1)**				0.11				0.13
2	531.42	9.06	1053.79	0.05	60.85	−457.96	579.66	0.82
3	654.38	33.61	1275.15	0.04	608.73	28.98	1188.48	0.04
3+	240.62	−332.30	813.55	0.41	49.33	−537.24	635.90	0.87
**Administrative support staff FTE per DC FTE**								
	184.68	−374.47	743.82	0.52				
**Clinical support staff FTE per DC FTE**								
	391.42	−380.26	1163.10	0.32				
**MCG (ref = 1a-High Complexity)**				0.18				0.4
MCG1b-High Complexity	−390.61	−927.17	145.96	0.15	−351.14	−872.80	170.52	0.19
MCG1c-High Complexity	−254.05	−779.45	271.35	0.34	−152.69	−709.36	403.99	0.59
MCG2-Medium Complexity	−427.83	−1058.51	202.85	0.18	−168.56	−794.08	456.95	0.60
MCG3-Low Complexity	−706.02	−1303.43	−108.60	0.02	−580.36	−1221.04	60.32	0.08

*CI* confidence interval, *LCI* lower confidence interval, *UCI* upper confidence interval, *VHA* Veterans Health Administration, *DC* doctor of chiropractic, *FTE* full-time equivalent, *MCG* medical complexity grouping

## Discussion

### Characteristics of the VHA chiropractor employee workforce

**Fig. 1:**
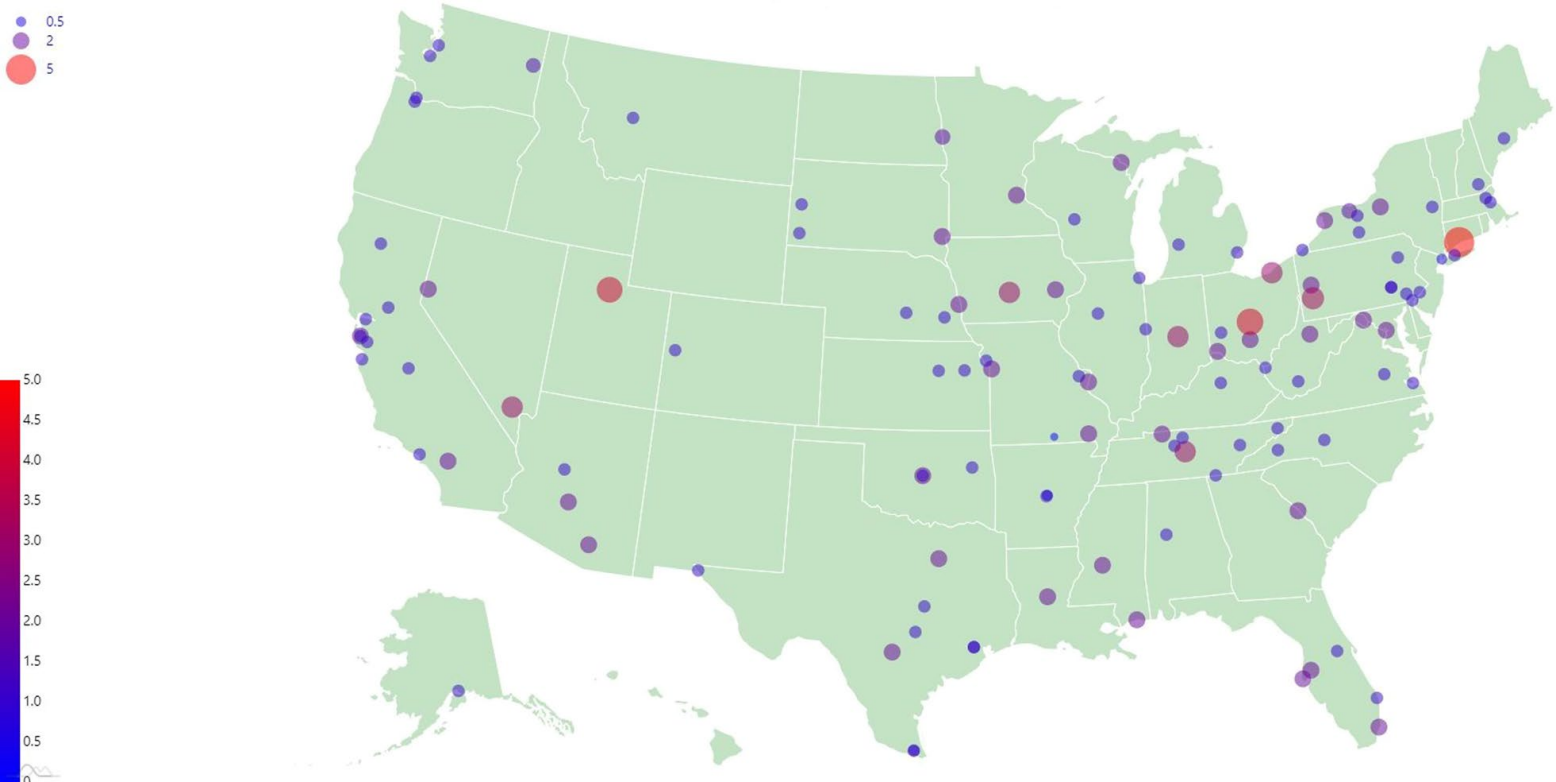
Geographic depiction of VHA chiropractor employee FTE (full-time equivalent)

These data show significant growth the VHA chiropractor employee workforce from FY2016-FY2019. Over the 4-year timeframe of this study, the number of chiropractor employees (including both full- and part-time positions) increased by 63.7%, which corresponds with a 72.7% increase in FTE. A depiction of the geographic distribution of VHA chiropractors at the end of FY2019 is presented in Fig. 1. A previous study showed that VHA chiropractic services has significantly expanded since their inception, with an average 18% increase per year in the number of Veterans receiving chiropractic services at VHA facilities from FY2005 to FY2015 [1]. This same study also reports on the number of chiropractor employees increased from 13 in FY2006 to 86 in FY2015 [1]. The current study, along with previous data, shows the VHA chiropractic program has continued to grow since its establishment through FY2019.

There are likely several reasons that the VHA chiropractic program has continued its trajectory of significant growth. The program is still relatively small in the VHA hospital system, which is the largest integrated healthcare system in the US and includes 170 medical centers and 1,074 outpatient facilities [4]. Continued demand for increased access to chiropractic care from Veterans and VHA physicians provides additional incentive for expansion [31]. There is also the increased use of both nonpharmacological therapies and complementary and integrative health approaches to pain management that has emerged in response to the nation’s opioid epidemic [32, 33] In 2016, the VHA conducted a State-of-the-Art Conference on nonpharmacological interventions for chronic musculoskeletal pain [15]. The recommendation from that conference was that VHA increase its delivery of evidence-based nonpharmacological approaches including chiropractic services, which is congruent with the recommendation of several clinical practice guidelines for common musculoskeletal spine conditions [11–13, 15, 34]. Legislation has also mandated the expansion of the VHA chiropractic program during this time [5]. It is likely that all of these factors have had some impact on the increased growth of the VHA chiropractic program.

The demographic composition of the VHA chiropractor employee workforce stayed relatively consistent from FY2016 to FY2019, although there are some indications that the profession may be trending to be more diverse. As of FY2019, the typical VHA chiropractor employee was male, white, and 45.9 years old with 5.2 years of VHA experience. Prior work reported in FY2015, the typical VHA chiropractor employee was the same age, male, and had slightly less VHA experience at 4.5 years [1]. This decreased VHA experience may be partially explained by the relative newness of the program and a workforce that had less time to accrue experience. The percentage of female employees increased in FY2019 while the percentage of white employees decreased. Veterans also accounted for 20.4% of chiropractor employees in FY2019, which was an increase from FY2016.

Compared to all US chiropractors, the VHA chiropractic workforce increase in the proportion of female chiropractors is consistent with an increasing proportion of female chiropractors within the US [26]. The VHA chiropractic workforce, at 25% female, slightly trails behind the total US chiropractic profession which is estimated as 32% female [26]. The VHA chiropractor workforce has a lower percentage of white employees than the total US chiropractor population at 91%, suggesting that the VHA chiropractic workforce may represent a more racially and ethnically diverse subset of the US chiropractor population [26]. It is beyond the scope of this paper to assess if VHA chiropractor demographic characteristics are associated with any patient or system level outcomes, yet we note similarities between chiropractor demographics and those of the VHA patient population, which is 98% male, 78% white, and median age of 64 years [35].

In FY2019, the mean annual salary of a VHA chiropractor was $104,959 which is significantly higher than the 2019 median chiropractor salary reported by the US Bureau of Labor Statistics of $70,340 [36]. The general US chiropractic workforce is working largely in other chiropractors offices (63%) or self-employed (31%), making careers within larger integrated healthcare systems a relative rarity [36]. The higher salary and low percentage of US chiropractors employed in integrated medical settings likely gives these healthcare systems increased leverage at selecting highly qualified chiropractors.

### Productivity of the workforce

As of FY2019, the mean productivity measure of a VHA chiropractor employee is 3040 wRVUs/FTE and the median was 2890 wRVUs/FTE. These results represent an initial benchmarking of VHA chiropractor productivity, since VHA uses actual internal historical performance to establish productivity targets. Such targets are established at the aggregate specialty or discipline level and are updated at least every 2 years or as significant changes warrant. By default, specialty provider group practice productivity that falls within the interquartile range (25th to 75th percentile) of prior VHA internal experience is considered an acceptable range of productivity. VHA does not compare productivity measures between different provider types, since different specialties have differing RVU values for their most commonly performed procedures, which results in differences in average levels of productivity values across specialties. However, the productivity values we report are largely consistent with the productivity measure of private hospital chiropractors, with a mean of 3050 wRVUs/FTE and median of 2677 wRVUs/FTE in FY2019 [37]. We are aware of no other published research on the magnitude of clinical productivity of chiropractor employees. One study of private sector medical facilities that implemented chiropractor services found from stakeholder interviews that the clinical productivity of chiropractic services was thought to be one measure of success of the implementation [38]. While the measures of clinical productivity was not presented in that paper, it does highlight the importance of monitoring clinical productivity for healthcare systems implementing chiropractic services [38].

In our results, the facility complexity measure, number of unique patients seen by one provider, total number of encounters, years served in the system, and provider age were significantly associated with productivity individually. However, only three variables remained significant after adjusting for other covariates (Table 3). Providing care in a MCG3 complexity facility was associated with marginally lower productivity than MCG1a facilities (coef = −580.36, *p* = 0.08) after adjusting for demographic characteristics (sex, age group, years served) and facility characteristics (number of patients seen, total encounters, DCs in the facility). Provider age is strongly correlated with productivity in the univariable model, especially for 60+ years vs. < 40 years and the effect trend and significance preserve after adjusting for other factors. Lastly, providing care at facilities with 3 DCs is associated with significantly higher productivity than facilities with only 1 DC (coef = 608.73, *p* = 0.04) after adjusting for other factors. However, we are cautious in interpreting that finding since the effect of number of DCs is not linear, and the sample size of facilities having more than 3 DCs is small. By comparison, many factors that were not associated with productivity in our results have been shown to be associated with productivity in VHA for other healthcare disciplines [39, 40]. We feel this is most likely due to the relative newness of chiropractic services in VHA, in that such services have not yet reached a stable level of system penetration. Expansion seen during our study period has included new facilities starting chiropractic clinics, and existing clinics adding chiropractors, thus the workforce is still evolving to a maturation point. Furthermore, we expect that the tendency of increased productivity with an increased number of DCs at a facility in particular is worthy of future study.

### Limitations

There are a number of limitations due to the retrospective analysis of administrative data, which is known to be frequently incomplete. This study is a cross-sectional examination, which inherently limits our ability to make any claims about causation. As a preliminary analysis, we made no attempts to define the relationship among our covariates beyond assessing for potential correlations. It remains unclear which variables are confounders when accessing for VHA chiropractor productivity. This study only included VHA employees categorized as chiropractors in VHA’s Human Resources Employee database, thus chiropractor who were not employees (for instance contractors) and those who were employees but may have been miscategorized in the databases were not included. We could not impute data to compensate for missing values in our multivariable model, but had to drop only 2 subjects due to missing data. Labor mapping of the VHA employees may be inaccurate and may impact both the clinical FTE of the chiropractor used in the calculation to determine chiropractor productivity and the assignment of clinical and administrative support staff to the chiropractic clinic. While clinical and administrative support staff assigned to any VHA clinics may be inaccurate, VHA chiropractic clinics tend to be smaller than other healthcare disciplines, frequently with only 1 or 2 employees, and as such portions of support staff assigned to the chiropractic clinic may suffer from more inaccuracy than clinics that do not share support staff. In presenting the productivity measure, the clinical productivity only takes into account procedures that were coded by CPT® codes and had an assigned wRVU value. This may undercount services that the chiropractic workforce provided but did not code for. The productivity measure presented here is only one measure of clinical productivity, and it does not take into account the quality of the care delivered.

## Conclusions

Provider characteristics and productivity metrics of the VHA chiropractor employee workforce are presented. Productivity of VHA chiropractors is similar to the private sector. Of the characteristics studied, only a higher facility complexity measure, presence of 3 chiropractor employees at a facility, and older age of the providers had a significant association with clinical productivity. These results may be relevant to future modeling of chiropractor personnel in VHA and other healthcare systems.

## Data Availability

To maximize protection security of veterans’ data while making these data available to researchers, the US Department of Veterans Affairs (VA) developed the VA Informatics and Computing Infrastructure (VINCI). VA researchers must log onto VINCI via a secure gateway or virtual private network connection (VPN), and use a virtual workspace on VINCI to access and analyze VA data. By VA Office of Research and Development policy, VINCI does not allow the transfer of any patient-level data out of its secure environment without special permission. Researchers who are not VA employees must be vetted and receive “without compensation” (WOC) employee status to gain access to VINCI. For questions about data access, contact the study lead (Kelsey.Corcoran@va.gov) or the VA Office of Research and Development (VHACOORDRegulatory@va.gov).

## Abbreviations

VHA: Veterans Health Administration
FY: Fiscal year
wRVUs: Work relative value units
OPES: VHA Office of Productivity, Efficiency, and Staffing
FTE: Full-time equivalent
CPT®: Current Procedural Terminology®
MCG: Medical complexity grouping
DC: Doctor of Chiropractic
